# Outpatient Management of COVID-19 With Monoclonal Antibody Therapy in a Young Renal Transplant Patient

**DOI:** 10.7759/cureus.17672

**Published:** 2021-09-03

**Authors:** Bilal Malik, Atefeh Kalantary, Kamal Rikabi, Basel Abdelazeem, Arvind Kunadi

**Affiliations:** 1 Internal Medicine, McLaren Health Care/Michigan State University, Flint, USA; 2 Internal Medicine, McLaren Flint/Michigan State University, Flint, USA

**Keywords:** covid-19, solid organ transplant, immunocompromised patient, monoclonal antibodies, renal transplant

## Abstract

At baseline, solid organ transplant recipients are at an increased risk for infectious complications due to the complex immunosuppressive regimens. The available data in renal transplant patients who contract coronavirus disease 2019 (COVID-19) demonstrates dangerously high mortality rates (33%) in those who require hospitalization and/or ICU level care. Interestingly, the data for transplant patients who do not require hospitalization shows significantly lower mortality (3%) despite being on an immunosuppressive regimen. We present the case of a young male patient with a history of renal transplant who tested positive for COVID-19; he was mildly symptomatic with cough, sinusitis, and headache, was worked up as an outpatient, and treated as an outpatient with bamlanivimab monotherapy with no adjustment to his immunosuppressive regimen. This case aims to highlight the possibility of safely managing mild cases of COVID-19 in solid organ transplant patients receiving immunosuppression as an outpatient with monoclonal antibody (mAb) therapy.

## Introduction

At baseline, solid organ transplant recipients are at an increased risk for infectious complications due to the complex immunosuppressive regimens required to keep their transplant from rejection. Data from across the world have demonstrated an increased mortality rate in renal transplant patients who have been infected with coronavirus disease 2019 (COVID-19) and required hospitalization [1]. Khairallah et al. (2021) reported a mortality rate as high as 33% in the studies and registries reviewed during their research, which was mainly attributed to respiratory failure associated with the virus [1]. Thaunat et al. (2020) conducted a study reviewing the mortality over the course of the pandemic in the French renal transplant population. Upon reviewing the data, they discovered that the excess mortality in patients following the onset of the COVID-19 pandemic was attributable directly to deaths related to COVID-19 infection, with 44% of deaths in this population from March to June 2020 being attributed to COVID-19 [2]. Further data from the European Renal Association (ERA) database confirmed these trends, with a mortality rate as high as 45% in renal transplant patients who required intensive care unit (ICU) level care [3]. In the same study by Hilbrands et al. (2020), an interesting dichotomy was evident - patients who did not require hospitalization did significantly better despite also being transplant recipients. Their mortality rate was as low as 3% [1,3].

We present the case of a young male patient with a history of renal transplant who tested positive for COVID-19, was mildly symptomatic with cough, sinusitis, and headache, was worked up as an outpatient, and treated as an outpatient with bamlanivimab monotherapy with no adjustment to his immunosuppressive regimen. This case aims to highlight the possibility of safely managing mild cases of COVID-19 in solid organ transplant patients receiving immunosuppression as an outpatient with guideline-directed monoclonal antibody (mAb) therapy.

## Case presentation

Our patient was a 21-year-old male with a history of acute promyelocytic leukemia (APML) at four years of age, subsequent tumor lysis syndrome, and renal transplant who presented to the hospital after being evaluated by his primary care provider for mild cough, sinusitis, and headache after contact with a recently confirmed COVID-19 patient. At age four, the patient had developed what appeared to be a flu-like illness. His mother noted his nose bleeding and after some time, the patient also developed hematemesis. At this point, the patient was taken into hospital and, upon investigation, found to have APML. During the treatment for his APML, the patient developed tumor lysis syndrome which left him dependent on hemodialysis due to renal damage. He was managed for end-stage renal disease (ESRD) on hemodialysis for an additional five years before he was able to receive a renal transplant at the age of nine. Since his transplant, the patient has been on immunosuppressive therapy with tacrolimus, prednisone, and mycophenolate. He has not experienced any complications of immunosuppression and has been adherent to therapy.

On initial presentation, he complained of cough, sinusitis, and headache. He did not have any respiratory distress, gastrointestinal symptoms, anosmia, or muscle aches. His mother tested positive with COVID-19 the same day and she was his most likely source of infection. He consulted his primary care physician (PCP) and was evaluated with laboratory studies and a chest X-ray. His chest X-ray was normal, without any ground-glass opacities or distinct lobar infiltrates/consolidations. Laboratory studies included complete blood count, complete metabolic profile, D-dimer, procalcitonin, and ferritin. All studies were found to be within normal ranges, apart from the D-dimer, which was marginally elevated at 0.55 mcg/mL (reference range <0.50 mcg/mL). A COVID-19 test was performed and was positive, prompting his PCP to recommend bamlanivimab therapy as an outpatient in view of his high-risk status as a solid organ transplant recipient. He successfully received the bamlanivimab transfusion and was managed safely at home with no further deterioration. He did follow up periodically with his PCP via televisit and made a complete recovery with no alteration to his immunosuppressive regimen, no decline in his renal function, and no other complications.

## Discussion

In solid organ transplant recipients at or further than two years post-transplant, Dale et al. (2021) found their mortality rate to be 18%, compared to a global mortality rate of 2.2% in the general population [4,5]. Risk factors predisposing renal transplant recipients to COVID-19 are similar to those in the general population, including obesity, advanced age, multiple comorbid conditions, poor overall condition, and pulmonary disease [1]. A recent study by Fishman et al. (2020) suggested that progression of COVID-19 in solid organ transplant patients occurred more rapidly, with a greater rate of admission to the ICU overall [1,6]. Interestingly, an additional feature predisposing to higher mortality was identified within this population - the time since transplantation. The patients who were less than a year out from receiving their transplant appeared to be at a higher risk, though the data were limited in availability [1].

In December of 2020, the US Food and Drug Administration (FDA) approved bamlanivimab for the treatment of patients with mild to moderate COVID-19 who are at high risk for hospitalization including advanced age, immunosuppression, obesity, diabetes, chronic kidney disease such as our patient [7,8]. By August 2021, the US FDA advised against the use of bamlanivimab and etesevimab due to the development of resistance amongst certain variants (the Gamma and Beta variants) of COVID-19 circulating within the United States [9]. In the same August 2021 update, several novel agents were recommended in place of prior mAb medications. In patients who had mild cases of COVID-19 with a high risk of progression (i.e. with the risk factors outlined above), casirivimab plus imdevimab, or sotrovimab (monotherapy) are now recommended [9]. Due to the constantly evolving and dynamic landscape surrounding the COVID-19 pandemic, it has been essential to follow the most current guidelines in managing patients with COVID-19. Our patient was successfully managed with guideline-directed therapy during his time of presentation, but at present, newer recommendations (as outlined above) should be followed. Their specific application in immunocompromised patients, including solid organ transplant patients, remains to be evaluated further.

Despite our patient being mildly symptomatic, his history of organ transplantation placed him at higher risk relative to the general population. Bamlanivimab is a recombinant neutralizing human immunoglobulin-G1 (IgG1) kappa mAb which binds to the viral spike protein on its outer surface that functions to facilitate cell invasion. In the BLAZE-1 trial by Chen et al. (2020), a randomized, double-blind, placebo-controlled clinical trial, there was a moderate reduction in the hospitalization of 465 adults with mild to moderate COVID-19 symptoms after administration of bamlanivimab within three days of the first positive severe acute respiratory syndrome coronavirus 2 (SARS-CoV-2) viral test [10]. In this study, bamlanivimab successfully lowered viral load at day 11 of infection, lowered the frequency of hospitalization, and had an acceptable tolerability profile [7,10-12]. No specific study has been published on transplant patients to date, but off-label use of bamlanivimab in transplant centers had shown promising results prior to resistant variants becoming prominent [13].

Casirivimab plus imdevimab are IgG1 kappa and IgG1 lamda recombinant human mAbs, respectively, that bind to the spike protein receptor binding domain and prevent viral entry into host cells via human angiotensin converting-enzyme 2 (ACE2) receptors [14]. The recommendations for casirivimab plus imdevimab were made based on phase 3 results from the R10933-10987-COV-2067 study (clinical trial Identifier NCT04519437), which reported the primary outcome of COVID related hospitalization or death in 1.0% and 3.2% (p=0.0024) of patients in the casirivimab plus imdevimab (n=736) and placebo groups (n=748), respectively [9]. The study demonstrated an absolute risk reduction (ARR) of 2.2% and a relative risk reduction (RRR) of 70% between the groups as outlined above. Sotrovimab also functions in a similar manner, binding to the spike protein on the virus. It is also a recombinant human IgG1 kappa mAb but does not competitively inhibit human ACE2 receptor binding. Sotrovimab inhibits a presently unidentified step in the process of fusion of the virus with the host cell membrane (after attachment but before fusion, per FDA data) [15]. The recommendations for its use are based on phase 3 COMET-ICE trial (clinical trial Identifier NCT04545060), which reported the primary endpoint of the proportion of patients who were hospitalized or died of any cause by day 29 [9]. These endpoint events occurred in 1% (n=291) and 7% (n=292) of patients within the sotrovimab and placebo groups (p=0.002), respectively [9]. The study demonstrated an absolute risk reduction (ARR) of 6% and a relative risk reduction (RRR) of 85% between the groups as outlined above.

The review article by Khairallah et al. (2021) provides an excellent flow diagram outlining major classifications and conceptualization of management of renal transplant patients who tested positive for COVID-19 [1]. Definitions and recommendations are summarized below in Table 1. Asymptomatic patients were recommended to consider decreasing the dosing of their antimetabolite agent.

**Table 1:** Summary of the definitions and management recommendations for each level of COVID-19 severity in renal transplant patients, as per Khairallah et al. (2021). [1]

	Mild	Moderate	Severe
Definition	lack of respiratory distress (i.e. tachypnea) well maintained saturations (>95% on pulse oximetry) negative chest roentgenogram	Positive roentgenogram findings maintained oxygen saturations >94% No extreme tachypnea (respiratory rate <30 breaths per minute)	Desaturation (<94% on pulse oximetry) significant tachypnea (respiratory rate >30 breaths per minute) despite supplemental oxygen Those requiring mechanical ventilation
Suggested intervention	commencement of low dose steroid therapy (if not already on steroids), maintenance of calcineurin inhibitors (CNI)/mammalian target of rapamycin inhibitors (mTORi) at the lowest possible therapeutic dose, cessation of antimetabolite drugs, and use of approved monoclonal antibody therapy.	similar to the mildly symptomatic category, except patients required high dose steroids and could also be tried on Remdesivir if acceptable by local guidelines.	Cessation of all immunosuppression was recommended along with high dose steroid therapy and Remdesivir, per local guidelines.

The RECOVERY trial, a randomized control trial involving 2104 patients receiving dexamethasone compared to 4321 patients receiving usual care, demonstrated a significantly decreased 28-day mortality in patients requiring respiratory support that received corticosteroids compared to placebo. These results were not applicable to those who did not require respiratory support (17.8% vs. 14.0%; rate ratio, 1.19; 95% CI, 0.91 to 1.55) [16,17]. In patients requiring mechanical ventilation or non-invasive oxygen therapy, there was a significant decrease in the incidence of death ([29.3% vs. 41.4%; rate ratio, 0.64; 95% CI, 0.51 to 0.81], [23.3% vs. 26.2%; rate ratio, 0.82; 95% CI, 0.72 to 0.94], respectively). Our patient did not require respiratory support, oxygen, or mechanical ventilation throughout his course of COVID-19 infection, and thus, was not a candidate for further steroid therapy on top of his baseline immunosuppressive regimen.

In general, there is limited data available to draw conclusions from for the management of transplant patients experiencing COVID-19 compared to the general population. Most guidelines support standard clinical practice for the treatment of COVID-19 in transplant recipients based on expert opinion [13,16]. Importantly, the COVID-19 pandemic has led to a constantly evolving landscape with regards to recommendations and legal authorizations to utilize certain agents. In a written communication by Denise M. Hinton (Chief Scientist, FDA, USA) on April 16, 2021, the FDA revoked the emergency use authorization for bamlanivimab monotherapy. The same communication further elucidated that the use of bamlanivimab remained under the authorization for use in conjunction with etesevimab as dual therapy at that time. Our patient was managed with bamlanivimab monotherapy as per the recommendations prior to these changes and the changes made in August 2021.

## Conclusions

Solid organ transplant patients are at an increased risk of morbidity and mortality when hospitalized with COVID-19. We presented the case of a young male with a history of solid organ (renal) transplant after being diagnosed with APML, developing tumor lysis syndrome, and becoming dialysis-dependent early in his childhood. He was diagnosed with COVID-19 and successfully managed as an outpatient with bamlanivimab monotherapy prior to official CDC recommendation changes in view of resistant variants emerging. Further studies are needed to form the foundation for any concrete recommendations regarding thresholds for inpatient vs outpatient management, treatment modalities, and the alteration of immunosuppressive regimens. At present, the CDC recommends against the use of bamlanivimab and etesevimab in patients with mild to moderate COVID-19 in the United States due to emerging resistance patterns. Novel agents including casirivimab plus imdevimab and sotrovimab are now being used in their place under emergency use authorization within the United States.
